# Retained energy in lactating beef cows; effects on maintenance energy requirement and voluntary feed intake

**DOI:** 10.1093/tas/txac120

**Published:** 2022-08-25

**Authors:** Emma A Briggs, Amanda L Holder, Megan A Gross, Alexandra N Moehlenpah, Jared D Taylor, R R Reuter, Andrew P Foote, Carla L Goad, David L Lalman

**Affiliations:** Department of Animal and Food Sciences, Oklahoma State University, Stillwater, OK 74078, USA; Department of Animal and Food Sciences, Oklahoma State University, Stillwater, OK 74078, USA; Department of Animal and Food Sciences, Oklahoma State University, Stillwater, OK 74078, USA; Department of Animal and Food Sciences, Oklahoma State University, Stillwater, OK 74078, USA; Department of Veterinary Pathobiology, Oklahoma State University, Stillwater, OK 74078, USA; Department of Animal and Food Sciences, Oklahoma State University, Stillwater, OK 74078, USA; Department of Animal and Food Sciences, Oklahoma State University, Stillwater, OK 74078, USA; Department of Statistics, Oklahoma State University, Stillwater, OK 74078, USA; Department of Animal and Food Sciences, Oklahoma State University, Stillwater, OK 74078, USA

**Keywords:** efficiency, maintenance, milk yield, milk composition, residual gain

## Abstract

The objectives of these experiments were to determine the relationship between maintenance requirements and energy partitioned to maternal tissue or milk production in limit-fed Angus cows and to determine the relationship between retained energy during the lactation period to dry-period voluntary forage intake (VDMI). Twenty-four mature fall-calving Angus cows were used in a 79-d study during late lactation to establish daily metabolizable energy required for maintenance (ME_m_). Cows were individually fed daily a mixed diet (2.62 Mcal MEl/kg, 18.2% crude protein) to meet energy and protein requirements of 505 kg beef cows producing 8.2 kg milk daily. If cow BW changed by ±9 kg from initial BW, daily feed intake was adjusted to slow BW loss or reduce BW gain. Milk yield and composition were determined on 3 occasions throughout the study. Maintenance was computed as metabolizable energy intake minus retained energy assigned to average daily maternal tissue energy change, average daily milk energy yield, and average daily energy required for pregnancy. After calves were weaned, cows were fed a low-quality grass hay diet (8.2% crude protein, 65% NDF) and VDMI was measured for 21 days. Lactation maintenance energy was 83% the default value recommended by NASEM (2016. Nutrient Requirements of Beef Cattle: Eighth Revised Edition.) for lactating Angus cows. Increasing lactation-period retained energy (decreasing BW loss and increasing milk energy yield) was associated with lower maintenance energy requirements (*P* < 0.01; *R*^2^ = 0.92). Increased residual daily gain during lactation was associated with lower lactation maintenance energy requirements (*P* = 0.05; *R*^2^ = 0.17). Post-weaning VDMI was not related to late-lactation milk energy production, although sensitive to lactation period BCS and BW loss. These results contradict previous reports, suggesting that maintenance requirements increase with increasing milk yield.

## INTRODUCTION

The cow/calf sector uses 74% of the total feed energy required to produce one pound of carcass weight (Rotz et al., 2019). Furthermore, the cow/calf sector accounts for 77% to 81% of enteric CH_4_ emissions per unit of carcass weight (Baber et al., 2018; Rotz et al., 2019). Therefore, improvements in energy utilization efficiency by the cow herd would result in both reduced cost of beef production and carbon footprint.

The maintenance requirement for energy is defined as the energy needed to achieve no net loss or gain of energy retained in the tissues of the animal’s body (NASEM, 2016). For perspective, average annual energy requirement for 550-kg beef cows producing 8 kg of milk at peak lactation is about 4,875 Mcal NE_m_, with 73% partitioned to maintenance, 10% to pregnancy, and 17% to lactation (NASEM, 2016). Similarly, Ferrell and Jenkins (1987) reported 70% to 75% of total annual energy expenditure is used for maintenance. These authors also noted that variation in maintenance requirement is greater than variation in requirements for growth, gestation, or lactation.

Over the last several decades, most beef breeds have been selected for increased growth, carcass weight and mature size (Capper, 2011; Kuehn and Thallman, 2016). At the same time, some breeds have aggressively selected for increased calf weaning weight through milk expected progeny differences (Kuehn and Thallman, 2016). Numerous reports suggest a positive relationship between maintenance energy requirement and genetic capacity for milk yield, mature size, and growth (Ferrell and Jenkins, 1984; Ferrell and Jenkins, 1987; Solis et al., 1988; Laurenz et al., 1991). However, these studies were structured to determine differences in maintenance requirements among breeds and breed crosses rather than within a breed, i.e. it is difficult to separate potential effects of breed vs. milk yield and other traits. In a recent study with sheep (Yang et al., 2020), authors suggested that long-term selection for increased productivity may be responsible for a 40% increase in net energy required for maintenance compared with recommendations of AFRC (1993), which were developed using data that is now over 40 years old. In the current energy system for beef cows (NASEM, 2016), productivity (or performance) can be quantified as energy retained in the form of body tissue, milk, and conceptus tissue. The objective of this experiment was to determine the relationship between maintenance requirements and energy partitioned to maternal tissue or milk production in limit-fed Angus cows using a long-term feeding approach. A second objective was to determine the relationship between retained energy during the lactation period to dry period voluntary forage intake.

## MATERIALS AND METHODS

All procedures and protocols were approved by Oklahoma State University Animal Care and Use Committee (#AG-17-26). Experiments were conducted at the Range Cow Research Center near Stillwater, OK. Twenty-four fall-calving cows and their calves were used in two consecutive experiments to evaluate the relationship of energy partitioned to milk production and maternal tissue to maintenance energy requirements. From January 7 (day –18) to March 26 (79 days), cows were individually fed a total-mixed ration (TMR) at a rate approximating each cow’s daily energy requirements according to NASEM (2016). Subsequently, milk energy yield, maternal tissue energy change, and energy used for pregnancy were subtracted from daily metabolizable energy intake (MEI) to estimate maintenance requirements. Following weaning, a voluntary feed intake trial was conducted from June 12 to July 19 to determine dams’ voluntary feed intake (experiment 2) during the dry (nonlactating) period.

All cows were managed as a contemporary group prior to the initiation of the restricted feeding experiment (experiment 1). Cows calved during September and October 2018 while grazing native tallgrass prairie pastures. Dried distiller’s grains with solubles were fed at the rate of 1.5 kg/d throughout the calving season and the feeding rate was increased to 2.5 kg/d through November and December. The 7-d Co-Synch protocol (Stein et al., 2015) was initiated on November 8, followed by timed artificial insemination performed 10 days later. Cows were then exposed to a fertile bull for an additional 50 days. At 0700 hours on January 7, bulls were removed, cows were fed in the individual feeding facility for the first time, and pairs were subsequently transferred to one of two pens.

### Experiment 1: lactation performance and maintenance requirements

Cows and their calves were randomly assigned to one of the two pens (12 cows and 12 calves each). Each pen was 32.9 × 32.9 m, dirt-surfaced, and was equipped with: fence-line feed bunks, a windbreak on both north and south perimeters, an automatic livestock watering system (MiraFount A3465, Miraco Automatic Livestock Waterers, Grinnell, Iowa), and an 80-m^2^ creep feeding area equipped with two individual feed intake measurement units (C-Lock Inc, Rapid City, South Dakota). Pens were stocked to provide approximately 90 m^2^ surface area per cow–calf pair.

Cows were fed a TMR (Table 1) at 0700 hours daily in a stall barn equipped with individual feeding stanchions. Before feeding, calves were penned in the creep feeding area. Cows were then moved to the stall barn one pen at a time. The order pens were brought into the stall barn was rotated daily to minimize any potential confounding effect of time of feeding. Cows were loaded into the stalls individually and offered TMR, allowing approximately 1 h to consume their ration. They were then returned to their pen. At that time, creep area gates were opened to allow calves access to the entire pen and to the cows. Calves were fed the same TMR as the mature cows and had continual access to the creep area where individual feed intake units were located (Table 1). Feed was placed in the creep area intake units at 0730 hours each day and if necessary, again at 1600 hours to ensure calves had ad libitum access to feed with a minimum of 10% daily orts. Cows never had access to the creep feed area or its feed intake units.

**Table 1.** Ingredient and chemical composition of the diets in experiments 1 and 2

Item	Experiment 1	Experiment 2
Ingredient, % DM basis		
Bermudagrass hay	48.86	
Native grass hay	—	94.5
Corn distiller’s grains	25.45	
Rolled corn	16.55	
Liquid supplement	3.98^1^	5.5^2^
Soybean meal, 44% CP^2^	2.39	
Limestone	2.20	
Salt	0.56	
Chemical composition, DM basis		
CP^3^, %	18.2	8.2
NDF^3^, %	33.85	65.1
ADF^3^, %	19.6	42.4
TDN^4^, %	71.6	53.4
DE^5^, Mcal/kg	3.20	2.36
ME^6^, Mcal/kg	2.62	1.94

Liquid supplement (Quality Liquid Feeds, Dodgeville, WI) chemical composition, DM basis = 15% CP, 2.3% NaCl, 0.5% P, 0.9% Ca, 70,500 IU vitamin A/kg.

Liquid supplement chemical composition, DM basis = 42.1% CP, 2.75 Mcal ME/kg, 2.5% NaCl, 0.84% P, 0.72 % CA, 66,000 IU vitamin A/kg.

CP = crude protein, NDF = neutral detergent fiber, ADF = acid detergent fiber.

TDN = total digestible nutrients determined as DE/4.409 (NASEM, 2016).

DE = digestible energy, computed using the summative equation (NRC 2001) with modifications recommended by Weiss and Tebbe (2018). The contribution of NDF to DE was determined using 48 h in vitro NDF digestibility.

ME = metabolizable energy, calculated as DE × 0.82.

The Beef Cattle Nutrient Requirements Model (BCNRM; NASEM, 2016) was used to estimate the daily TMR allowance for each cow that would provide the amount of feed energy required to maintain body weight (BW) and support 8.2 kg daily milk production (Andresen et al., 2020) and pregnancy during late lactation (20.8 Mcal ME/d or 71 g/kg BW^0.75^ TMR, DM basis). For the first 18 days, cows were adapted to the TMR, limit-feeding strategy, and the feeding facility in the following manner. Starting on day –18, cows were fed 35% TMR and 65% chopped bermudagrass hay at the rate of 95 g/kg initial BW^0.75^ for 4 days. Subsequently, the feeding rate was reduced by 6 g/kg initial BW^0.75^ at 4-day intervals. At the same time, dietary proportion of TMR was increased and the hay was decreased by 16.25 percentage units at 4-day intervals. This resulted in cows being fed 100% TMR and 71 g/kg BW^0.75^ on the morning of day –`2. Cows and calves were weighed using a hydraulic squeeze chute equipped with electronic load cells (Tru-Test HD5T; Datamars, Mineral Wells, TX) and an electronic weigh scale indicator (Tru-Test XR5000; Datamars). The experimental period began on the morning of January 25 (day 0) and continued for 61 consecutive days.

Cow BW was recorded at 0700 hours prior to feeding on 10 days at approximate 6-day intervals, beginning on day 0 and continuing through day 61. Cows had ad libitum access to water throughout the experiment. Because cow BW was recorded at least 18 h after the previous day’s feeding event, our BW data represent shrunk BW (NASEM, 2016). Cow body condition score (BCS; 1 to 9, Wagner et al., 1988) was recorded on approximately 14-day intervals at the same time BW was recorded. Two experienced technicians recorded BCS, and these two scores were averaged within animal for each date. Daily feed allotment was adjusted by ≤0.45 kg DM if an individual’s BW fluctuated by ≥9 kg above or below initial (day 0) shrunk BW (Cooper-Prado et al., 2014). Subsequent adjustments (≤0.45 kg DM) were made if weight change continued to increase or decrease ≥9 kg above or below initial study shrunk BW.

Samples of TMR were collected weekly. Dry matter was determined by oven drying at 60 °C for 4 h. Dried samples were ground through a Wiley Mill grinder (Model-4, Thomas Scientific, Swedesboro, NJ, USA) using a 2-mm screen and later analyzed for concentrations of ash (combusted 6 h in a muffle furnace at 500 °C), CP (N×6.25; CN628, LECO Corporation, St. Joseph, MI, USA), neutral detergent fiber (aNDF, Van Soest et al., 1991) and acid detergent fiber (ADF, AOAC, 1990, #973.18) were analyzed using an ANKOM Delta Automated Fiber Analyzer (Ankom Tech Corp, Fairport, NY, USA). Neutral detergent fiber was assayed with alpha amylase and sodium sulfite. Both aNDF and ADF are expressed inclusive of residual ash. Fat content was determined utilizing the ether extract method (AOAC, 1990). The summative equation (NRC, 2001 with modifications recommended by Weiss and Tebbe, 2018) was used to determine digestible energy (DE) by multiplying the digestible masses of CP, NDF, fat, and nonfiber carbohydrate by their enthalpies (5.6, 4.2, 9.4, and 4.2, respectively; Weiss and Tebbe, 2018). The mass of digestible NDF was determined using 48-h in vitro digestibility (NRC, 2001). Feed consumed from days 0 through 61 was multiplied by feed ME (Mcal/kg) to determine the total feed energy consumed during the experimental period.

Linear or quadratic regression equations were calculated for each cow using BW and BCS regressed over time (Ferrell and Jenkins, 1996) and these equations were used to determine initial (day 0) and final (day 61) BW and BCS. Initial and final cow BW was adjusted to a non-pregnant basis retrospectively using subsequent calving season birth date and birth BW (NASEM, 2016). Cow BW, adjusted to a non-pregnant basis, was used to calculate average daily gain (ADG) and metabolic mid-point BW.

Total body energy for each cow was computed retrospectively for days 0 and 61 using the methods described by NASEM (2016). Briefly, equations first published by NRC (1996, 2000) use BCS to compute the proportion of empty BW that is fat and protein. Next, body protein and fat proportion are multiplied by empty BW to determine total body fat and total body protein. Finally, total body fat (kg) and total body protein (kg) are multiplied by their biological energy value (9.4 and 5.7 Mcal/kg, respectively). Calculated total body energy for day 0 was subtracted from calculated total body energy for day 61 to determine body net energy change (Mcal NEm). If BW loss occurred during the 61-day experimental phase, the loss in energy was multiplied by 0.8 to estimate Mcal NEm available for maintenance during mobilization (NASEM, 2016).

Milk yield was measured and milk samples were collected on days 5, 33, and 62 by the procedure described by Wiseman et al. (2019). On the day before milking, calves were removed from their dams at 1400 hours. Calves were not allowed access to creep feed during this period. At 2000 hours, calves were returned to their dams and were allowed to suckle until satiated. At the conclusion of the suckling period (2045 hours), calves were again removed from their dams. Milking began the next morning at 0500 hours allowing for an average 8.5-h separation. Cows were milked with a portable milk machine (Portable Vacuum Systems, Springville, UT). To determine milk composition, a subsample was taken, preserved with 2-bromo-2nitropropane-1,3-diol and shipped to the Heart of America Dairy Herd Improvement Association laboratory (Manhattan, KS). To adjust for differences in dam-calf separation time, rate of milk production (g/min) was determined by dividing milk yield (g) by separation time (min). The rate of production was then multiplied by 1,440 min to calculate 24-h milk yield (Yn). Milk energy concentration was calculated as (NASEM, 2016)


$$\begin{array}{l} E=\left(0.092\;\times \;MkFat\right)+\left(0.049\;\times \;MkSNF\right)-0.0569 \end{array}$$

where *E* is the energy content of milk (Mcal/kg), MkFat is milk fat content (%), and MkSNF is milk solids non-fat content (%). Daily net energy partitioned to milk (NE_l_, Mcal/day) was calculated as


$$\begin{array}{l} NE_{l}=Yn\times \;E \end{array}$$

The average of the three NE_l_ estimates were used to determine daily net energy partitioned to milk production.

Net energy required for pregnancy (NE_y_, Mcal/day) was calculated retrospectively using calf birth BW and calf birth date from the subsequent calving season as follows (NASEM, 2016):


$$\begin{array}{l} NE_{y}=\left[CBW\;\times \;\left(0.5855-0.0000996\;\times \;DP\right)\;\times \;e^{\left(0.03233\;\times \;DP-0.0000275\;\times \;DP^{2}\right)}\right]/1,000 \end{array}$$

where CBW is calf birth BW, kg, and DP is days pregnant. Metabolizable energy required for pregnancy (ME_y_, Mcal/d) was converted to an ME basis using the fixed partial efficiency of 0.13 (NASEM, 2016):


$$\begin{array}{l} ME_{y}=NE_{y}/0.13 \end{array}$$

Total retained energy (NE_r_) was obtained by summing energy partitioned to or produced by maternal tissue (NE_t_), NE_l_, and NE_y_. Retained energy from maternal tissue gain or loss and lactation were converted to an ME basis using the partial efficiency coefficient from the Garrett (1980) equation. Finally, maintenance energy requirement (ME_m_, Mcal/d) was estimated by subtracting retained energy pools (ME basis) from MEI:


$$\begin{array}{l} ME_{m}=\;MEI-ME_{t}-ME_{l}-ME_{y} \end{array}$$

### Experiment 2: voluntary forage intake

Following the conclusion of experiment 1, cows and their calves were turned out to pasture. On May 15, calves were weaned, and cows were palpated to determine pregnancy status. The voluntary forage intake study (experiment 2) was initiated on June 12 (day –21). Twenty-four gestating cows were assigned to similar dry lot pens as described for experiment 1. Three pens were used, each equipped with two individual feed intake units (C-Lock, Inc., Rapid City, South Dakota). The diet (8.2% CP, 1.94 Mcal ME/kg; DM basis) is shown in Table 1 and consisted of 94.5% (DM basis) chopped native tall-grass prairie hay and 5.5% (DM basis) sugarcane molasses-based liquid supplement shown in Table 1. The liquid supplement was sprayed onto the processed hay and thoroughly mixed. Subsequently, 5% (as-fed basis) water was sprayed onto the diet and thoroughly mixed prior to feeding. Cows were fed twice daily to maintain at least 10% daily orts in the feed intake units to ensure ad libitum access to feed. The intake units were stocked at 4 cows per feeder, i.e., 8 cows per pen. Weekly feed samples were collected and analyzed for chemical composition as previously described for experiment 1. Cows were adapted to the diet and feeding system for the first 21 days and daily feed intake was recorded for the following 21 days.

Body weights were recorded at 0700 hours on days –21, 0, 1, 20, and 21 using the same scale system described for experiment 1. Because cattle were provided access to feed on an ad libitum basis prior to and throughout the experiment, all weights were adjusted to a shrunk BW basis (BW × 0.96; NASEM, 2016). For each BW recorded, non-pregnant BW was calculated by subtracting the estimated BW of the conceptus as described for experiment 1 (NASEM, 2016). Fetal age was determined retrospectively based on calving date the following year. Non-pregnant BW was then used to determine ADG and metabolic mid-point BW.

### Statistical Analyses

Pearson correlation coefficients were calculated (SAS 9.4; SAS Inst. Inc., Cary, NC) to determine the relationships between late-lactation performance characteristics, energy partitioning, and subsequent nonlactating voluntary dry matter intake (VDMI). Dependent variables used to compute ME_m_ were investigated for multicollinearity using multiple linear regression and evaluating variance inflation factor, tolerance, and collinearity diagnostics (SAS 9.4; SAS Inst. Inc.). Forward stepwise linear regression was used to explore the influence of each of the four independent variables used to compute ME_m_. At each step, variables were chosen according to their contribution to the model’s coefficient of determination (*R*^2^). Residual average daily gain (RADG) was computed for each cow as the residual from mixed model regression (SAS 9.4; SAS Inst. Inc.) of shrunk BW average daily gain (SADG) on MEI, study-average BCS, and milk yield (kg/day). The average number of days each cow was pregnant during the trial was included as a random variable. The effects of time on calf feed intake, scaled to BW, were characterized using a spline regression model (NLIN procedure, SAS 9.4; SAS Inst. Inc.) to determine whether a break point in time existed, and if so, the slope of the two resulting regression lines.

## RESULTS AND DISCUSSTION

In experiment 1, mean days in milk was 177 ± 17 (Table 2). Late-lactation milk yield averaged 8.4 ± 1.23 kg/day while milk energy concentration averaged 0.70 ± 0.06 Mcal/kg. Mean daily milk energy yield did not differ by month (*P* = 0.21; 5.89 ± 0.9 Mcal/day; data not shown). Andresen et al. (2020) reported similar late-lactation milk yield in limit-fed mature cows from this herd, although in that study, greater milk fat concentration (3.8%) resulted in greater milk energy concentration (0.73 Mcal/kg). Considering cows in the current experiment had lower mean BCS, daily BW gain and daily MEI/kg BW^0.75^, lower milk energy concentration is not surprising. After experiment 1 was completed, three cows were determined to be nonpregnant. Data from these three cows remained in the data set with no adjustments for estimated weight change associated with fetal tissue and zero energy partitioned to NE_y_ (pregnancy). Mean estimated daily NE_y_ in pregnant cows was minimal, averaging 3.0% of total NE_r_.

**Table 2.** Summary statistics of production and feed intake traits for limit-fed Angus cows (*N* = 24)

Item^1^	Mean	Min	Max	SD
Avg DMI, kg/day	7.94	7.23	8.79	0.43
Avg DMI, g/kg BW^0.75^	74.8	71.1	78.7	2.04
Day 0 MEI, kcal/kg BW^0.75^	194.9	182.3	212.1	7.6
Day 61 MEI, kcal/kg BW^0.75^	196.4	180.7	217.4	9.4
Avg MEI, kcal/kg BW^0.75^	196.5	189.6	203.7	3.9
Day 0 BW, kg	506.5	426.1	562.7	35.8
Day 61 BW, kg	500.8	419.9	562.3	37.0
BW change, kg	-5.72	-31.0	16.8	11.9
SADG, kg/day	-0.09	-0.51	0.28	0.20
BCS	4.9	3.9	6.0	0.47
Avg days in milk	177.5	139	201	17.2
Milk yield, kg/day	8.4	6.9	13.2	1.23
Milk yield, g/kg BW^0.75^	79.3	63.3	118.3	12.1
Milk energy, Mcal/kg milk	0.70	0.58	0.82	0.06
Milk protein, %	2.95	2.39	3.62	0.30
Milk fat, %	3.61	1.23	5.40	0.72
Milk solids-not-fat, %	8.75	5.4	9.47	0.30
NE_l_, kcal/kg BW^0.75^	54.9	44.9	73.6	7.6
NE_t_, kcal/kg BW^0.75^	-5.0	-19.3	9.4	8.6
NE_y_, kcal/kg BW^0.75^	1.55	0	3.0	0.86
NE_r_, kcal/kg BW^0.75^	51.3	34.2	69.0	9.9
ME_m_, kcal ME/kg BW^0.75^	118.0	91.5	148.2	15.1

MEI = metabolizable energy intake; BW = study-average cow body weight adjusted for pregnancy; BCS = study-average body condition score; SADG = shrunk average daily gain; NE_l_ = net energy for lactation; NE_t_ = net energy provided by (weight loss) or partitioned to (weight gain) maternal tissue; NE_y_ = net energy for pregnancy; NE_r_ = total retained energy; ME_m_ = metabolizable energy for maintenance.

Although cows were initially assigned uniform calculated feed energy intake scaled to BW^0.75^, weight change associated with the adaptation period resulted in modest variation in day 0 calculated ME intake per kg BW^0.75^ (CV = 3.9%). Considering minimal mean BW change during the experimental period (–5.7 ± 11.9 kg), the BCNRM provided a reasonably accurate estimate of energy requirements to achieve BW stasis (on average) for this group of cows. Variation in BW change during the experimental period was expected due to potential differences in efficiency of feed conversion to DE, ME, and NE (NASEM, 2016), as well as differences in NE_l_ and NE_m_. In an effort to achieve BW stasis for each cow, adjustments in daily feed allowance were made when a cow’s BW gain or BW loss exceeded 9 kg. These adjustments were only marginally successful because there was a wide range in final calculated BW change (–31.0 to 16.8 kg). This is likely due to the combination of modest adjustments in daily feed allowance (≤0.45 kg of feed DM) combined with the experimental period being limited to 61 days. In fact, the first four cows requiring feed allowance adjustment did not meet the ± 9 kg criteria until day 27. Overall, daily feed allowance adjustments were made for 14 cows between days 27 and 54.

The BCNRM assumes equal efficiency of ME use for NE_m_, NE_t_, NE_l_, and NE_y_. Efficiency of ME use is computed using diet ME concentration (Garrett et al., 1980) or using a fixed value of 0.6 (NASEM, 2016). To compute NE_m_, we first converted NE_r_ to an ME basis using a fixed value for *K*_m_ (0.654; Garrett, 1980). Subsequently, ME_r_ was subtracted from MEI to compute ME_m_. The *k*_m_ value generated by the Garrett (1980) equation did not differ substantially from that reported by Reynolds and Tyrrell, (2000; 0.64) and Freetly et al., (2006; 0.69) using primiparous beef cows. Patle and Mudgal, (1977) estimated *k*_m_ of 0.65 in Brown Swiss × Sahiwal crossbred lactating cows.

The resulting estimate of mean NE_m_ was 83% (77.1 kcal/kg SBW^0.75^) of the default value used for lactating Angus cows in the BCNRM (92.4 kcal NE_m_/kg BW^0.75^). Similarly, previous reports from this herd (Andresen et al., 2020; Wiseman et al., 2019) estimated NE_m_ requirements in limit-fed beef cows lower than the BCNRM default value. Freetly et al. (2006) and Trubenbach et al. (2019) also reported lower estimates of NE_m_ when cows are limit fed an energy-dense diet.

As Freetly et al. (2019) described, maintenance requirements and efficiency of ME utilization for maintenance and (or) production are not independent. At the same level of MEI scaled to BW, increased NE_r_ leads to a lower estimate of NE_m_ when *k*_m_ is fixed. However, if NE_m_ is fixed, increased NE_r_ leads to an increased estimate of *k*_m_. Overall, default values for NE_m_ and *k*_m_ used in the BCNRM resulted in a reasonably accurate prediction of the amount of feed energy required for these cows. However, the lower estimate of NE_m_ could also indicate that *k*_m_ was underestimated by the Garrett (1980) equation. For example, increasing *k*_m_ to 0.80 results in the same NE_m_ used in the BCNRM for lactating Angus cows.

Mean, minimum, maximum, and standard deviation for performance and VDMI characteristics for experiment 2 are shown in Table 3. Late-gestation VDMI of this low-quality diet was considerably greater (13.8 ± 2.8 kg) than predicted by the model used in BCNRM (11.5 ± 0.55; NASEM 2016, Eq. 10-5). This equation is sensitive to cow BW and diet energy concentration. Previous feed restriction of an energy-dense diet, experiment 2 forage particle size (chopped), and added molasses-based liquid feed and water to forage in experiment 2 may contribute to excessive feed intake in this experiment.

**Table 3.** Summary statistics of cow performance and voluntary forage intake (*N* = 24), experiment 2

Item^1^	Mean	Min	Max	SD
Days pregnant	223	191	243	14.7
BW, kg	580.7	515.3	634.8	33.9
BCS	5.1	3.6	6.4	0.63
SADG, kg	0.32	-1.44	0.83	0.48
VDMI, kg/day	13.8	9.1	20.0	2.8
VDMI, g/kg BW^0.75^	117.2	79.8	182.9	24.3

Days pregnant = study-average days pregnant for pregnant cows (*n* = 21); BW = study-average shrunk body weight adjusted for fetal tissue weight; BCS = study-average body condition score; SADG = shrunk average daily gain adjusted for fetal tissue weight; VDMI = voluntary dry matter intake.

Pearson correlation coefficients for performance traits and energy partitioning are presented in Table 4. Cows with greater study-average SBW produced less NE_l_ (*r* = –0.44, *P* = 0.03). However, when SBW was adjusted for BCS according to NASEM (2016), there was no significant relationship with NE_l_ (*r* = –0.31, *P* = 0.14; data not shown).

**Table 4.** Pearson correlation coefficients between late-lactation body weight, body condition, weight gain, and energy partitioning (experiment 1) and nonlactating voluntary dry matter intake ( experiment 2)^1^

Item^2^	SBW	BCS	SADG	MEIv	NE_t_	NE_l_	NE_y_	ME_m_
BCS	0.31 0.14							
SADG	0.10 0.64	**0.64** **< 0.01**						
MEI	-0.27 0.21	0.32 0.12	0.23 0.28					
NE_t_	0.19 0.38	**0.40** **0.05**	**0.65** **< 0.01**	-0.17 0.43				
NE_l_	**-0.44** **0.03**	-0.31 0.14	**-0.40** **0.05**	**0.42** **0.04**	-0.26 0.22			
NE_y_	-0.05 0.83	0.12 0.59	-0.02 0.94	-0.29 0.17	0.10 0.65	-0.02 0.94		
ME_m_	0.11 0.63	-0.03 0.88	-0.20 0.35	0.11 0.60	**-0.72** **< 0.01**	**-0.43** **0.04**	-0.23 0.27	
VDMI	-0.14 0.51	-0.35 0.09	**-0.46** **0.02**	0.10 0.64	-0.27 0.20	0.30 0.15	-0.12 0.57	0.16 0.45

SBW = experiment 1 pregnancy-adjusted shrunk body weight, kg; BCS = expriment 1 body condition score; SADG = expriment 1 pregnancy-adjusted shrunk average daily gain, kg; MEI = expriment 1 metabolizable energy intake, kcal ME/kg BW^0.75^; NE_t_ = expriment 1 maternal tissue energy retained, Kcal/kg BW^0.75^; NE_l_ = expriment 1 milk energy retained, kcal/kg BW^0.75^; NE_y_ = expriment 1 pregnancy energy retained, kcal/kg BW^0.75^_;_ ME_m_ = expriment 1 energy required for maintenance, kcal ME/kg BW^0.75^; VDMI = expriment 2 nonlactating voluntary dry matter intake, g/kg BW^0.75^.

For each cell, the top number is the correlation coefficient (*r*), and the bottom number is the *P*-value. Coefficients with *P* ≤ 0.05 are bolded.

There was a moderate negative correlation (Table 4; *r* = –0.40, *P* = 0.05) between SADG and NE_l_, suggesting that milk energy production was antagonistic to a cow’s ability to maintain BW. This is not surprising because initial daily feed allocation was based on cow BW with no adjustment for milk yield. Secondly, the length of the experimental period did not allow time for feed intake adjustments to completely offset the impact that increased milk yield had on maternal tissue BW change. Rahnefeld et al. (1990) also reported greater BW and condition loss with increased milk yield. Similarly, Mondragon et al. (1983) reported that increasing milk yield during the first and second parity contributed to negative energy balance, reducing cow BW and condition at the time of calving in the subsequent parity. However, when energy change associated with maternal tissue was adjusted for BW and BCS (NE_t_), there was no relationship between estimated maternal tissue energy change and milk energy produced (NE_l_; *r* = –0.26, *P* = 0.22). While the correlation of mean BCS during late lactation to NE_l_ was not significant (*r* = –0.31; *P* = 0.14), the correlation between BCS recorded during experiment 2 and NE_l_ was negative (*r* = –0.40; *P* = 0.05; data not shown). Together, these results suggest that increasing yield of milk energy was associated with greater late-lactation BW loss.

Even though MEI adjustments were modest, there was a moderate positive correlation between MEI and NE_l_ (Table 4; *r* = 0.42, *P* = 0.04). However, there was no relationship between MEI and NE_t_. These results suggest that additional feed energy was primarily partitioned to milk production and that milk energy yield is highly sensitive to feed energy availability in agreement with Jenkins and Ferrell (1994) and Lalman et al. (2000).

**Figure 1.**
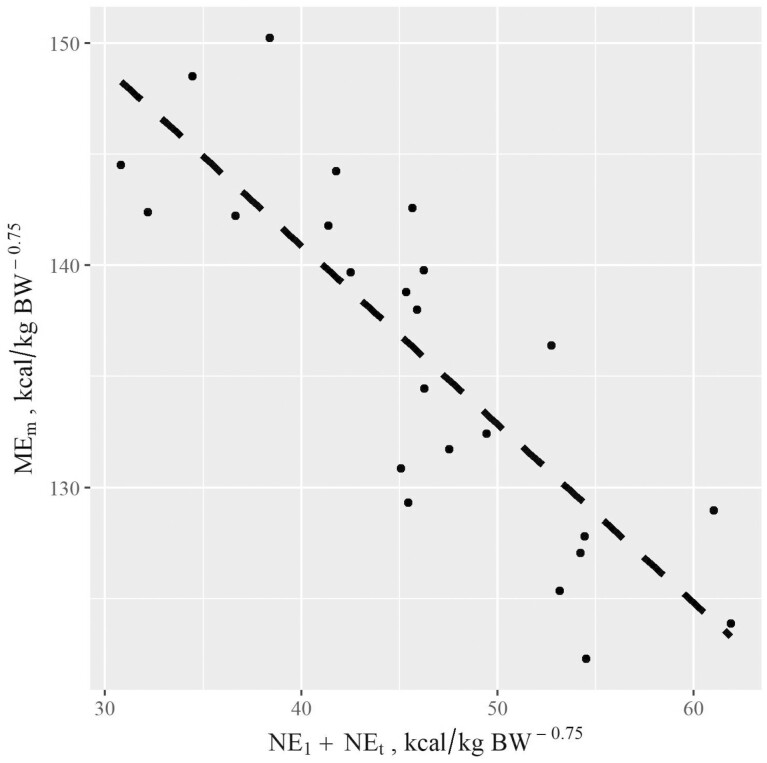
There was a negative relationship of net energy partitioned to milk (NE_l_) and maternal tissue energy change (NE_t_) to metabolizable energy used for maintenance (ME_m_) when beef cows were limit fed a mixed concentrate/forage diet; ME_m_, kcal/kg BW^0.75^ = 182.4 (6.7) – 1.572 (0.11) * NE_t_ – 1.321 (0.12) * NEl (*R*^2^ = 0.92; all variables in the model *P* < 0.001). Cows with lower maintenance requirements had more energy to allocate to milk and maternal tissue energy.

In this experiment, ME_m_ was computed using four predictor variables: MEI, NE_t_, NE_l_, and NE_y_. There was no evidence of multicollinearity among the four variables with tolerance ≥0.74 and variance inflation factor ≤1.34. Stepwise model selection revealed that most of the variation in ME_m_ was accounted for by NE_t_ (partial *R*^2^ = 0.517) and NE_l_ (partial *R*^2^ = 0.407) with minimal influence of MEI (partial *R*^2^ = 0.069) and NE_y_ (partial *R*^2^ = 0.006). The relationship of ME_m_ to NE_l_ + NE_t_ is shown in Figure 1. As expected, when maintenance requirement was adjusted for BW, feed energy supplied, milk energy yield, and energy required for pregnancy, cows that lost more BW had greater estimated maintenance requirement. However, increased milk energy production was associated with lower ME_m_. Under these conditions, even though cows producing greater milk energy lost more BW, the estimated energy contained in the modest BW loss was not sufficient to offset the retained energy gained with increased milk energy yield. These results oppose previously reported studies which have suggested that as milk yield potential increases, there is an associated increase in maintenance requirements (Ferrell and Jenkins, 1984; Jenkins and Ferrell, 1994; Montaño-Bermudez et al., 1990). Reynolds and Tyrrell (2000) suggested that cattle’s maintenance requirement may not be directly related to production levels. As mentioned previously, we assumed constant *k*_m_ to compute ME_m_. It is possible that some or all the improved efficiency associated with decreased weight loss and increased milk yield is the result of improved efficiency of ME utilization (*k*_m_; Freetly et al., 2019).

For the past few decades, residual feed intake (RFI) has been used as a genetic selection tool to improve feed efficiency as it is relatively independent from mature body size, unlike other phenotypic measures of feed efficiency such as the gain-to-feed ratio (Basarab et al., 2011; Castro Bulle et al., 2007). Basarab, et al. (2011), reported a tendency for a positive correlation between RFI and maintenance energy requirements (0.421; *P* = 0.10), suggesting that low RFI animals have more net energy available for production. In our study, RFI was not calculated for experiment 1 because cows’ daily feed allowance was controlled. Rather, a multiple regression equation using MEI, BCS, and kg milk yield was used to predict SADG (*R*^2^ = 0.56) and subsequently to calculate RADG.

Residual average daily gain was negatively correlated to MEm (*r* = –0.41; *P* = 0.049; data not shown) and is characterized by the following equation:


$$\begin{array}{l} MEm,\;\;\;kcal\;\;\;ME\;kg\;\;\;BW^{0.75}=118.0\pm 2.9-46.4\;\pm 22.3\times RADG,\;\;\;kg \end{array}$$

Several studies report higher partial efficiency of ME use for growth in low-RFI cattle (Nkrumah et al., 2006; Cantalapiedra-Hijar et al., 2018). In dairy cattle, low-RFI cows showed a greater efficiency of converting feed energy to net energy as well as required less net energy for maintenance than high-RFI cows with similar BW (Vandehaar et al., 2016). Together, these studies and the results of the current experiment suggest that cattle with high RADG or low RFI may have lower maintenance requirements and (or) increased efficiency of ME use.

**Figure 2.**
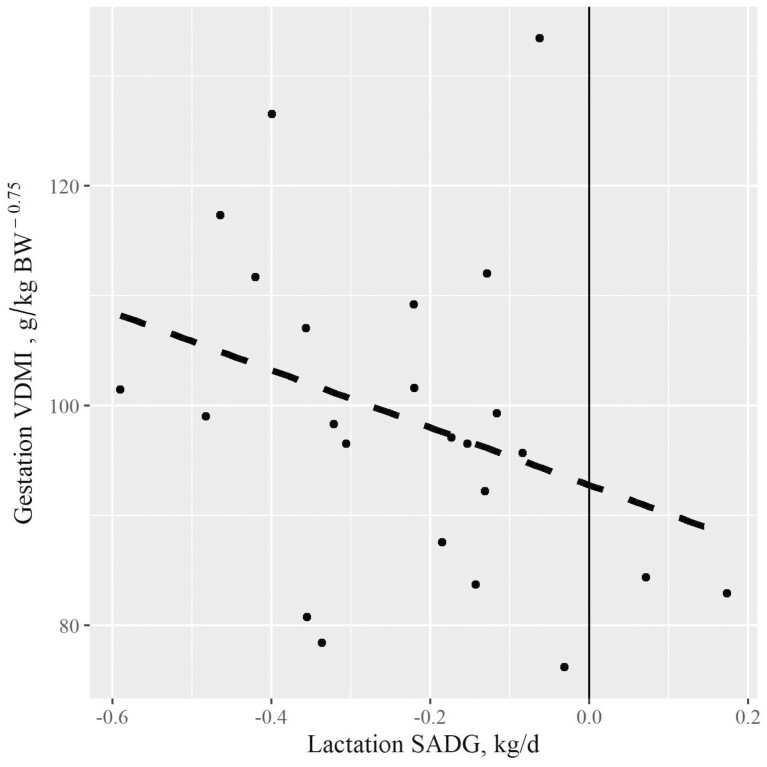
Relationship of lactation-period shrunk average daily gain (SADG) to gestation-period voluntary dry matter intake (VDMI); VDMI, g/kg BW^0.75^ = 102.4 (4.4) – 49.4 (20.4) × SADG, kg/day (*R*^2^ = 0.21; SADG coefficient *P* = 0.02).

The relationship of late-lactation SADG to non-lactating VDMI is shown in Figure 2. The linear coefficient indicates that each 1 kg SADG BW loss is associated with 49.4 g/kg BW^0.75^ increase in VDMI during the dry period (experiment 2). The influence of previous plane of nutrition on VDMI in beef cows is not well documented. Fox et al. (1988) estimated DMI increased by 2.7% for each one percent decrease in body fat composition for growing cattle within the range of 21.3% to 31.5% body fat (Fox et al., 1988). Using this relationship, the BCNRM uses initial BCS to predict body fat composition (NASEM, 2016) and subsequently, to adjust feed intake for previous plane of nutrition. Holder (2022) reported that DMI increased by 1.5% for each 1% reduction in body fat composition when beef cows consumed low-quality hay and by 2.4% when beef cows consumed a concentrate/forage mixed diet. Assuming each unit of BCS change is associated with 3.8% change in body fat and that each unit BCS is associated with 0.0714 × SBW change (NASEM, 2016), the linear regression coefficient for VDMI equates to 8.3% increase in VDMI for each 1% loss in body fat composition. This estimate is substantially greater than that reported by Fox et al. (1988) and Holder (2022). We are not aware of other reports relating VDMI to previous BW change. Mean estimated body fat composition was 18.6% (± 1.8) for these cows during experiment 1, and below the minimum in the growing cattle data set used by Fox et al. (1988; 21.3%). Additionally, this relationship should be viewed with caution because the range in BCS during experiment 1 was limited to only 2.1 BCS units (3.9 to 6.0).

### Calf Performance

**Figure 3.**
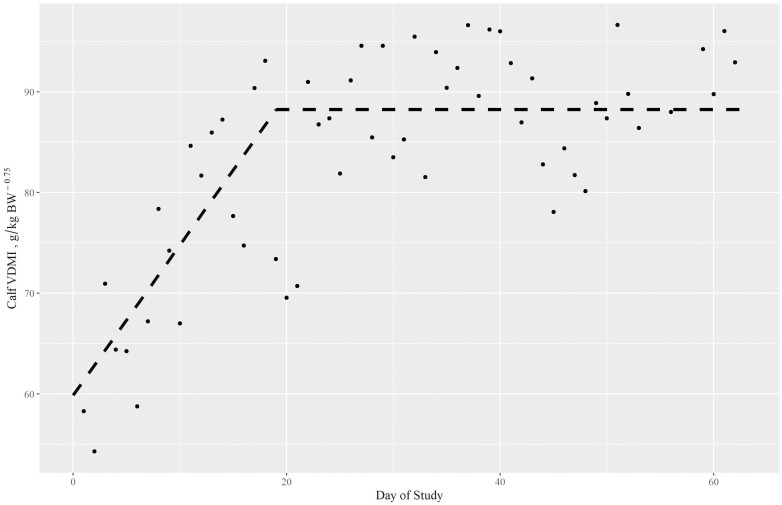
Observed mean daily calf voluntary dry matter intake (VDMI; circles), and linear – plateau regression model (dashed line). VDMI, g/kg BW^0.75^ = 58.3 (3.3) + 1.576 (0.286) × day; plateau (x0) = 19.03 (2.04) and VDMI, g/kg BW^0.75^ = 88.25 (*R*^2^ = 0.61).

Little data are available characterizing creep feed intake of nursing beef calves in the drylot (Lusby et al., 1976). The mean, standard deviation, and range for calf BW, ADG, and VDMI for experiment 1 are shown in Table 5. A linear-plateau spline model fit the mean daily VDMI data set with a breakpoint at day 19 ± 2.0 (Figure 3). Prior to the breakpoint, feed intake increased at the rate of 1.58 ± 0.29 g/kg BW^0.75^ per day. After day 19, feed intake stabilized at 88.25 g/kg BW^0.75^ per day. Calves were first exposed to the creep area and provided ad libitum access to TMR beginning on day –18. The only previous exposure to concentrate feed would have been the opportunity to compete for a portion of the cows’ concentrate supplement (fed on the ground in the pasture) prior to the initiation of the experiment in early January. Creep feed consumption data was not recorded during the adaptation period (days –18 to 0). Therefore, assuming acclimation to the creep area, feeders, and feed was occurring during the adaptation period, feed intake scaled to BW increased through day 19, for a total of 37 days, prior to stabilizing.

**Table 5.** Summary statistics of calf performance and voluntary feed^1^ intake (*N* = 24), experiment 1

Item^2^	Mean	Min	Max	SD
BW, kg	203.8	173.7	235.8	18.9
ADG, kg	1.63	1.32	1.96	0.16
VDMI, kg/d	4.67	3.69	5.59	0.58

Calves had ad libitum access to the same TMR fed to cows (Table 1).

BW = study-average body weight; ADG = average daily gain; VDMI = voluntary dry matter intake.

Increasing feed intake by nursing calves is expected during late lactation because dams’ daily milk yield declines during this time while calf BW increases (Wood, 1967; Boggs et al., 1980; NASEM, 2016). Tedeschi et al. (2009) found that alfalfa hay intake in calves receiving different amounts of reconstituted milk replacer was influenced by milk DMI and calf BW. In our study, mean daily milk energy yield did not differ by month. Therefore, assuming calf milk energy intake is equivalent to milk energy production, milk energy intake scaled to BW declined while feed intake scaled to BW increased over time. In this experiment where calves only had access to milk and TMR, after day 19, calf VDMI averaged 2.27 ± 0.17 g/kg BW. Boggs et al. (1980) also reported rapid increase in forage intake from May through September in spring-born nursing beef calves. In their study, late lactation (September) grazed forage VDMI was similar, averaging 2.2 g/kg BW.

Previous studies have documented a negative relationship between forage intake and milk intake in grazing, nursing beef calves (Lusby et al., 1976; Boggs et al., 1980; and Ansotegui et al., 1991) and drylot, early-weaned dairy calves (Abdelsamei et al., 2005). In the current experiment, there was no relationship between dam’s mean daily milk energy production and calf VDMI (*P* = 0.17; data not shown) of a mixed concentrate/forage diet. It is unknown whether this discrepancy is due to the creep diet (48% concentrate feeds) and (or) confinement housing compared to forage diets and pasture housing in the studies of Lusby et al. (1976)Boggs et al. (1980), and Ansotegui et al. (1991). Potential sources of error in our data include limited (3) measurements of milk production, differences between estimates of dam milk yield and calf milk consumption, or cross-nursing between pairs while housed in dry-lot pens.

## APPLICATIONS

Limit feeding a mixed forage/concentrate diet during late lactation resulted in maintenance energy requirement 83% of the default value recommended by NASEM (2016). Under these conditions, maternal tissue energy change, as estimated by BCS and BW change, explained more of the variation in ME_m_ than did NE_l_. Maintenance requirement estimates were lower in cows with greater total energy recovery (NE_t_ + NE_l_) in contrast to previous reports conducted across different breeds. More work is necessary to determine if increased retained energy per unit of MEI is due to lower maintenance, increased efficiency of ME utilization, or both. Finally, post-weaning VDMI was not related to late-lactation milk energy production, although sensitive to lactation period BCS and BW loss.

## References

[CIT0001] Abdelsamei, A., D. Fox, L. Tedeschi, M. Thonney, D. Ketchen, and J. Stouffer. 2005. The effect of milk intake on forage intake and growth of nursing calves. J. Anim. Sci. 83:940–947. doi:10.2527/2005.834940x. 15753351

[CIT0002] Agricultural and Food Research Council (AFRC). 1993. Energy and protein requirements of ruminants; An advisory manual prepared by the AFRC Technical Committee on Responses to Nutrients.CAB Int., Oxford, UK.

[CIT0003] Andresen, C. E., A. W. Wiseman, A. McGee, C. Goad, A. P. Foote, R. Reuter, and D. L. Lalman. 2020. Maintenance energy requirements and forage intake of purebred vs. crossbred beef cows. Transl. Anim. Sci. 4:1182–1195. doi:10.1093/tas/txaa008. PMC7201081 32705009

[CIT0004] Ansotegui, R. P., K. M. Havstad, J. D. Wallace, and D. M. Hallford. 1991. Effects of milk intake on forage intake and performance of suckling range calves. J. Anim. Sci. 69:899–904. doi:10.2527/1991.693899x. 2061260

[CIT0005] AOAC. 1990. Association of Official Analytical Chemists, Official Methods of Analysis., 15th ed. Arlington, VA: AOAC.

[CIT0006] Baber, J. R., J. E. Sawyer, and T. A. Wickersham. 2018. Estimation of human-edible protein conversion efficiency, net protein contribution, and enteric methane production from beef production in the United States. Transl. Anim. Sci. 2(4):439–450. doi:10.1093/tas/txy086. 32704726 PMC7200519

[CIT0007] Basarab, J. A., M. A. Price, J. L. Aalhus, E. K. Okine, W. M. Snelling, and K. L. Lyle. 2011. Residual feed intake and body composition in young growing cattle. Can. J. Anim. Sci. 83(2):189–204. doi:10.4141/A02-065. https://cdnsciencepub.com/doi/abs/10.4141/A02-065.

[CIT0008] Boggs, D. L., E. F. Smith, R. R. Schalles, B. E. Brent, L. R. Corah, and R. J. Pruitt. 1980. Effects of milk and forage intake on calf performance. J. Anim. Sci. 51:550–553. doi:10.2527/jas1980.513550x.

[CIT0009] Cantalapiedra-Hijar, G., M. Abo-Ismail, G. E. Carstens, L. L. Guan, R. Hegarty, D. A. Kenny, M. McGee, G. Plastow, A. Relling, and I. Ortigues-Marty. 2018. Review: biological determinants of between-animal variation in feed efficiency of growing beef cattle. Animal 12:s321–s335. doi:10.1017/S1751731118001489. 30139392

[CIT0010] Capper, J. L. 2011. The environmental impact of beef production in the United States: 1977 compared with 2007. J. Anim. Sci. 89:4249–4261. doi:10.2527/jas.2010-3784. 21803973

[CIT0011] Castro Bulle, F. C. P., P. V. Paulino, A. C. Sanches, and R. D. Sainz. 2007. Growth, carcass quality, and protein and energy metabolism in beef cattle with different growth potentials and residual feed intakes1,2. J. Anim. Sci. 85:928–936. doi:10.2527/jas.2006-373. 17178805

[CIT0012] Cooper-Prado, M. J., N. M. Long, M. P. Davis, E. C. Wright, R. D. Madden, J. W. Dilwith, C. L. Bailey, L. J. Spicer, and R. P. Wettemann. 2014. Maintenance energy requirements of beef cows and relationship with cow and calf performance, metabolic hormones, and functional proteins. J. Anim. Sci. 92:3300–3315. doi:10.2527/jas.2013-7155. 24902599

[CIT0013] Ferrell, C. L., and T. G. Jenkins. 1984. Energy utilization by mature, nonpregnant, nonlactating cows of different types. J. Anim. Sci. 58:234–243. doi:10.2527/jas1984.581234x. 6698902

[CIT0014] Ferrell, C. L., and T. G. Jenkins. 1987. Influence of biological types on energy requirements. In: Proceedings of Grazing Livestock Nutrition Conference; Jackson, WY; p. 1–7.

[CIT0015] Ferrell, C. L., and T. G. Jenkins. 1996. Relationships between body condition score and empty body weight, water, fat, protein, and energy percentages in mature beef cows of diverse breeds. J. Anim. Sci. 74:245.8778105

[CIT0016] Fox, D. G., C. J. Sniffen, and J. D. O’Connor. 1988. Adjusting nutrient requirements of beef cattle for animal and environmental variations. J. Anim. Sci. 66:1475–1495.

[CIT0017] Freetly, H. C. 2019. Fiftieth Anniversary of the California Net Energy System Symposium: What are the energy coefficients for cows? Transl. Anim. Sci 3:969–975. doi:10.1093/tas/txz024. 32704861 PMC7200495

[CIT0018] Freetly, H. C., J. A. Nienaber, and T. Brown-Brandl. 2006. Partitioning of energy during lactation of primiparous beef cows1. J. Anim. Sci. 84:2157–2162. doi:10.2527/jas.2005-534. 16864877

[CIT0019] Garrett, W. N. 1980. Energy utilization by growing cattle as determined in 72 comparative slaughter experiments. In: Energy Metabolism. Butterworth-Heinemann. p. 3–7.

[CIT0020] Holder, A. L., M. A. Gross, A. N. Moehlenpah, C. L. Goad, M. Rolf, R. S. Walker, J. K. Rogers, and D. L. Lalman. 2022. Effects of diet on feed intake, weight change, and gas emissions in beef cows. J. Anim. Sci. 100:1–9. doi:10.1093/jas/skac257. PMC9527298 35952719

[CIT0021] Jenkins, T. G., and C. L. Ferrell. 1994. Productivity through weaning of nine breeds of cattle under varying feed availabilities: I. Initial evaluation. J. Anim. Sci. 72:2787–2797. doi:10.2527/1994.72112787x. 7730170

[CIT0022] Kuehn, L. A., and R. M. Thallman. 2016. Beef Improvement Federation Annual Meeting & Symposium, June 14–17, 2016, Hilton Garden Inn, Manhattan, Kansas.

[CIT0023] Lalman, D. L., J. E. Williams, B. W. Hess, M. G. Thomas, and D. H. Keisler. 2000. Effect of diet energy on milk production and metabolic hormones in thin, primiparous beef heifers. J. Anim. Sci. 78:530–538. doi:10.2527/2000.783530x. 10764058

[CIT0024] Laurenz, J. C., F. M. Byers, G. T. Schelling, and L. W. Greene. 1991. Effects of season on the maintenance requirements of mature beef cows. J. Anim. Sci. 69(5):2168–2176. doi:10.2527/1991.6952168x. 2066326

[CIT0025] Lusby, K. S., D. F. Stephens, and R. Totusek. 1976. Effects of milk intake by nursing calves on forage intake on range and creep intake and digestibility in drylot. J. Anim. Sci. 43:1066–1071. doi:10.2527/jas1976.4351066x.

[CIT0026] Mondragon, I., J. W. Wilton, O. B. Allen, and H. Song. 1983. Stage of lactation effects, repeatabilities and influences on weaning weights of yield and composition of milk yield in beef cattle. Can. J. Anim. Sci. 63:751−761.

[CIT0027] Montaño-Bermudez, M., M. K. Nielsen, and G. H. Deutscher. 1990. Energy requirements for maintenance of crossbred beef cattle with different genetic potential for milk. J. Anim. Sci. 68:2279–2288. doi:10.2527/1990.6882279x. 2401650

[CIT0028] NASEM. 2016. Nutrient Requirements of Beef Cattle: Eighth Revised Edition. Available from: https://www.nap.edu/catalog/19014/nutrient-requirements-of-beef-cattle-eighth-revised-edition

[CIT0029] Nkrumah, J. D., E. K. Okine, G. W. Mathison, K. Schmid, C. Li, J. A. Basarab, M. A. Price, Z. Wang, S. S. Moore. 2006. Relationships of feedlot feed efficiency, performance, and feeding behavior with metabolic rate, methane production, and energy partitioning in beef cattle. J. Anim. Sci. 84(1):145–153. doi:10.2527/2006.841145x. PMID: 16361501. 16361501

[CIT0030] NRC 200 National Research Council. 2000. Nutrient Requirements of Beef Cattle: Seventh Revised Edition: Update 2000. Washington, DC: The National Academies Press. doi:10.17226/9791.

[CIT0031] National Research Council (NRC). 1996. Nutrient requirements of beef cattle, 7th Revised Edition: Update 1996. Washington, DC: The National Academies Press. doi:10.17226/9791.

[CIT0032] NRC. 2001. Nutrient Requirements of Dairy Cattle. 7th rev. ed. Washington, DC: The National Academies Press. 38386771

[CIT0033] Patle, B. R., and V. D. Mudgal. 1977. Utilization of dietary energy for maintenance, milk production and lipogenesis by lactating crossbred cows during their midstage of lactation. Br. J. Nutr. 37:23–33. doi:10.1079/bjn19770004. 557330

[CIT0034] Rahnefeld, G. W., G. M. Weiss, and H. T. Fredeen. 1990. Milk yield and composition in beef cows and their effect on cow and calf performance in two environments. Can. J. Anim. Sci. 70(2):409–423. doi:10.4141/cjas90-053.

[CIT0035] Reynolds, C. K., and H. F. Tyrrell. 2000. Energy metabolism in lactating beef heifers. J. Anim. Sci. 78:2696–2705. doi:10.2527/2000.78102696x. 11048936

[CIT0036] Rotz, C. A., S. Asem-Hiablie, S. Place, and G. Thoma. 2019. Environmental footprints of beef cattle production in the United States. Agric. Sys. 169:1–13. doi:10.1016/j.agsy.2018.11.005.

[CIT0037] Solis, J. C., F. M. Byers, G. T. Schelling, C. R. Long, L. W. Greene. 1988. Maintenance requirements and energetic efficiency of cows of different breed types. J. Anim. Sci. 66(3):764–773. doi:10.2527/jas1988.663764x. PMID: 3378932. 3378932

[CIT0038] Stein, D., B. Freking, and B. Whitworth. 2015. Synchronizing heats in beef cows and heifers. In: Lalman D. L., and D. Doye, editors, Beef cattle manual. 7th ed. Stillwater, OK: Oklahoma State University Cooperative Extension; p. 289–296.

[CIT0039] Tedeschi, L. O., D. G. Fox. 2009. Predicting milk and forage intake of nursing calves. J. Anim. Sci. 87(10):3380–3391. doi:10.2527/jas.2009-2014. Epub 2009 Jul 2. PMID: 19574576. 19574576

[CIT0040] Trubenbach, L. A., T. A. Wickersham, L. N. Bierschwale, J. C. Morrill, J. R. Baber, and J. E., Sawyer. 2019. Limit feeding as a strategy to increase energy efficiency in intensified cow–calf production systems. Trans. Anim. Sci. 3(2):796–810 doi:10.1093/tas/txz039. PMC7200819 32704847

[CIT0041] Vandehaar, M., L. Armentano, K. Weigel, D. M. Spurlock, R. Tempelman, and R. Veerkamp. 2016. Harnessing the genetics of the modern dairy cow to continue improvements in feed efficiency. J. Dairy Sci. 99(6):4941–4954. doi:10.3168/jds.2015-10352. 27085407

[CIT0042] Van Soest, P. J., J. B. Robertson, and B. A. Lewis. 1991. Methods for dietary fiber, neutral detergent fiber and nonstarch polysaccharides in relation to animal nutrition. J. Dairy Sci. 74:3583–3597. doi:10.3168/jds.S0022-0302(91)78551-2. 1660498

[CIT0043] Wagner, J. J., K. S. Lusby, J. W. Oltjen, J. Rakestraw, R. P. Wettemann, and L. E. Walters. 1988. Carcass composition in mature Hereford cows: estimation and effect on daily metabolizable energy requirement during winter. J. Anim. Sci. 66:603–612. doi:10.2527/jas1988.663603x. 3378920

[CIT0044] Weiss, W. P., and A. W. Tebbe. 2018. Estimating digestible energy values of feeds and diets and integrating those values into net energy systems. Trans. Anim. Sci. 3:953–961. doi:10.1093/tas/txy119. PMC7200586 32704859

[CIT0045] Wiseman, A. W., M. Redden, A. McGee, C. Spencer, R. R. Reuter, G. W. Horn, and D. L. Lalman. 2019. Effects of timing of weaning on energy utilization in primiparous beef cows and post-weaning performance of their progeny. J. Anim. Sci. 97:1198–1211. doi:10.1093/jas/skz019. 30668783 PMC6396231

[CIT0046] Wood, P. 1967. Algebraic model of the lactation curve in cattle. Nature 216:164–165. doi:10.1038/216164a0.

[CIT0047] Yang, C. T., C. M. Wang, Y. G. Zhao, T. B. Chen, A. Aubry, A. W. Gordon, T. Yan. 2020. Updating maintenance energy requirement for the current sheep flocks and the associated effect of nutritional and animal factors. Animal 14(2):295–302. ISSN 1751-7311. doi:10.1017/S1751731119002064. 31554532

